# Supplementary material for: The Hepatokine RBP4 Links Metabolic Diseases to Articular Inflammation
Source: Antioxidants (Basel). 2024 Jan 19;13(1):124. doi: 10.3390/antiox13010124 (PMC10812991; doi:10.3390/antiox13010124)
Supplement: Supplementary file 1 [file antioxidants-13-00124-s001.zip › antioxidants-2775519-supplementary figures.pdf]

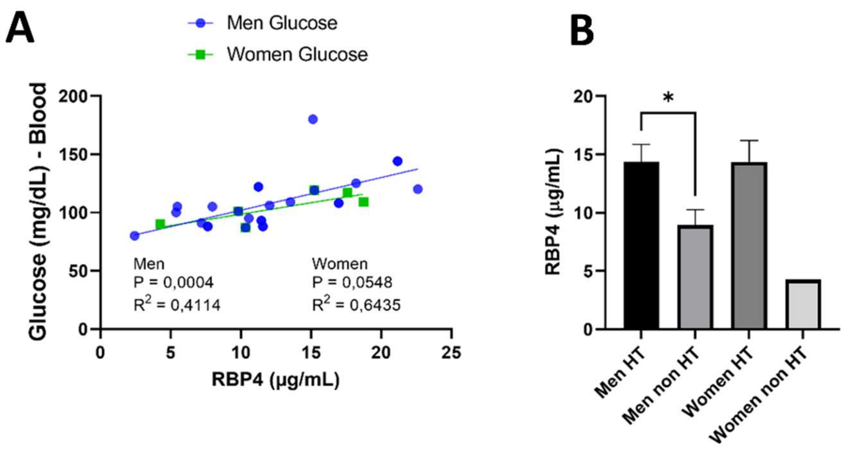

Supplementary Figure S1: Correlations for RBP4 levels tested by an ELISA assay on synovial fluid with blood glucose levels of patients obtained from their medical records and separated by sex. In men (n = 21), the correlation remains significative while in women (n = 6) is close to significance (A). When separating our patients by its hypertension diagnosis and sex, hypertense men have higher RBP4 levels, on the other side, we have just one sample of non-hypertensive women which does not allow us to perform the correspondent test (B), * p < 0.05.

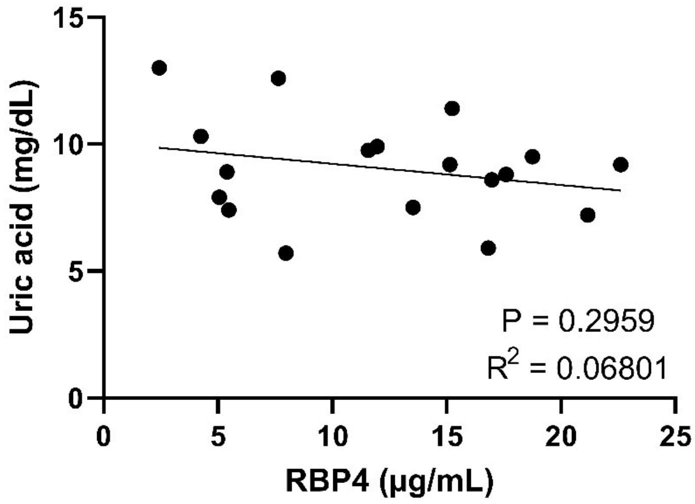

Supplementary Figure S2: Correlations for RBP4 levels tested by an ELISA assay on synovial fluid with uric acid levels of patients obtained from their medical records. There is not a significant correlation between both variables.
