# Supplementary material for: The Hepatokine RBP4 Links Metabolic Diseases to Articular Inflammation
Source: Antioxidants (Basel). 2024 Jan 19;13(1):124. doi: 10.3390/antiox13010124 (PMC10812991; doi:10.3390/antiox13010124)
Supplement: Supplementary file 1 [file antioxidants-13-00124-s001.zip › Supplementary Table S1.pdf]

Supplementary Table S1. Primers used for both mouse and human for all the RT-PCRs performed.

Mouse primers			
**Gene**	**Symbol**	**Forward primer**	**Reverse primer**
Interleukin 6	IL6	AAGAAATGATGGATGCTACC	GAGTTTCTGTATCTCTCTGAAG
Vascular cell adhesion molecule	VCAM	ACTGATTATCCAAGTCTCTCC	CCATCCACAGACTTTAATACC
Intercellular Adhesion Molecule 1	ICAM	CAGTCTACAACTTTTCAGCTC	CACACTTCACAGTTACTTGG
C-C Motif Chemokine Ligand 2	CCL2	CAAGATGATCCCAATGAGTAG	TTGGTGACAAAAACTACAGC
Nicotinamide Phosphoribosyltransferase	NAMPT	CAAGAGATTGTAGAGGGAATG	TCTCGGGTTAACTTCTGTAG
Nitric Oxide Synthase 2	NOS2	CATCAACCAGTATTATGGCTC	TTTCCTTTGTTACAGCTTCC
Retinol binding protein 4	RBP4	AATGGTTACTGTCAAAGCAG	AATAGAGATGAAGACCGGATG
Hypoxanthine Phosphoribosyltransferase 1	HPRT	AGGGATTTGAATCACGTTTG	TTTACTGGCAACATCAACAG
Matrix Metallopeptidase 1	MMP1	CAAGTCCGCTATTTCAAAGG	ACATGTGGTCAACAAAGAAG
Matrix Metallopeptidase 3	MMP3	CTTTAGAGGGAGAAAATTC	CATCATCATAACTCCACACG
Matrix Metallopeptidase 9	MMP9	CTTCCAGTACCAAGACAAAG	ACCTTGTTCACCTCATTTTG

Human primers			
**Gene**	**Symbol**	**Forward primer**	**Reverse primer**
Interleukin 6	IL6	GCAGAAAAAGGCAAAGAATC	CTACATTTGCCGAAGAGC
Vascular cell adhesion molecule	VCAM	ACTTGATGTTCAAGGAAGAG	TCCAGTTGAACATATCAAGC
Intercellular Adhesion Molecule 1	ICAM	ACCATCTACAGCTTTCCG	TCACACTTCACTGTCACC
C-C Motif Chemokine Ligand 2	CCL2	AGACTAACCCAGAAACATCC	ATTGATTGCATCTGGCTG
Nicotinamide Phosphoribosyltransferase	NAMPT	CTAATGGCCTTGGGATTAAC	TCCAGTGTAACAAAATTCCC
Retinol binding protein 4	RBP4	CCAGAAAGGAAATGATGACC	CTTACAATCTTCTGCGCTTC
Hypoxanthine Phosphoribosyltransferase 1	HPRT	ATAAGCCAGACTTTGTTGG	ATAGGACTCCAGATGTTTCC
Matrix Metallopeptidase 1	MMP1	AAAGGGAATAAGTACTGGGC	CAGTGTTTTCCTCAGAAAGAG
Matrix Metallopeptidase 3	MMP3	GCAGTTAGAGAACATGGAG	ACGAGAAATAAATTGGTCCC
Matrix Metallopeptidase 9	MMP9	AAGGATGGGAAGTACTGG	GCCCAGAGAAGAAGAAAAG
Chemokine (C-X-C motif) ligand 1	CXCL1	AACCGAAGTCATAGCCACAC	CACCAGTGAGCTTCCTCCTC
Chemokine (C-X-C motif) ligand 2	CXCL2	CCAAACCGAAGTCATAGCCAC	CTCCTTCAGGAACAGCCACC
Chemokine (C-X-C motif) ligand 8	CXCL8	CCACCGGAAGGAACCATCTC	TTCTCAGCCCTCTTCAAAAACT
Chemokine (C-X-C motif) ligand 12	CXCL12	CAGCGACGGGAAGCCC	GTCCTTTTTGGCTGTTGTGCT
Beta-actin	ACTB	CACAGAGCCTCGCCTTTGC	CCACCATCACGCCCTGG
