# Supplementary material for: The Hepatokine RBP4 Links Metabolic Diseases to Articular Inflammation
Source: Antioxidants (Basel). 2024 Jan 19;13(1):124. doi: 10.3390/antiox13010124 (PMC10812991; doi:10.3390/antiox13010124)
Supplement: Supplementary file 1 [file antioxidants-13-00124-s001.zip › Supplementary Table S2.pdf]

Supplementary Table S2. Summary of the ten best results of the molecular docking, showing the different molecular interactions and their values together with a final score. Results are sorted from best to worse.

Rank	Conformation	Electrostatics	Desolvation	Van de Waals	Total
1	1089	-26.82	-11.39	40.965	-34.663
2	9761	-2.716	-35.838	64.735	-32.080
3	164	-21.208	-11.412	9.707	-31.649
4	621	-12.543	-25.773	81.316	-30.185
5	2154	-19.775	-13.079	36.503	-29.203
6	8672	-11.493	-18.650	12.732	-28.870
7	8171	1.521	-37.182	71.976	-28.463
8	5027	-6.405	-24.281	31.853	-27.501
9	6341	-22.029	-10.423	58.914	-26.560
10	8626	-3.512	-32.986	102.654	-26.233
